# Core Microbiome of Medicinal Plant *Salvia miltiorrhiza* Seed: A Rich Reservoir of Beneficial Microbes for Secondary Metabolism?

**DOI:** 10.3390/ijms19030672

**Published:** 2018-02-27

**Authors:** Haimin Chen, Hongxia Wu, Bin Yan, Hongguang Zhao, Fenghua Liu, Haihua Zhang, Qing Sheng, Fang Miao, Zongsuo Liang

**Affiliations:** 1 College of Life Sciences, Northwest A&F University, Yangling 712100, China; chenhm@zstu.edu.cn (H.C.); hhzhang@zstu.edu.cn (H.Z.); 2 College of Life Sciences, Zhejiang Sci-Tech University, Hangzhou 310018, China; wuhx@zstu.edu.cn (H.W.); yanb@zstu.edu.cn (B.Y.); csheng@zstu.edu.cn (Q.S.); 3 Tianjin Tasly Holding Group Co., Ltd., Tianjin 300410, China; zhaohongguang@tasly.com (H.Z.); liufh@tasly.com (F.L.)

**Keywords:** seed-associated microbiome, 16S rRNA and ITS2 gene amplicons, Illumina sequencing, diversity, PICRUSt, *Salvia miltiorrhiza* Bge

## Abstract

Seed microbiome includes special endophytic or epiphytic microbial taxa associated with seeds, which affects seed germination, plant growth, and health. Here, we analyzed the core microbiome of 21 *Salvia miltiorrhiza* seeds from seven different geographic origins using 16S rDNA and ITS amplicon sequencing, followed by bioinformatics analysis. The whole bacterial microbiome was classified into 17 microbial phyla and 39 classes. Gammaproteobacteria (67.6%), Alphaproteobacteria (15.6%), Betaproteobacteria (2.6%), Sphingobacteria (5.0%), Bacilli (4.6%), and Actinobacteria (2.9%) belonged to the core bacterial microbiome. Dothideomycetes comprised 94% of core fungal microbiome in *S. miltiorrhiza* seeds, and another two dominant classes were Leotiomycetes (3.0%) and Tremellomycetes (2.0%). We found that terpenoid backbone biosynthesis, degradation of limonene, pinene, and geraniol, and prenyltransferases, were overrepresented in the core bacterial microbiome using phylogenetic examination of communities by reconstruction of unobserved states (PICRUSt) software. We also found that the bacterial genera *Pantoea, Pseudomonas*, and *Sphingomonas* were enriched core taxa and overlapped among *S. miltiorrhiza*, maize, bean, and rice, while a fungal genus, *Alternaria*, was shared within *S. miltiorrhiza*, bean, and Brassicaceae families. These findings highlight that seed-associated microbiomeis an important component of plant microbiomes, which may be a gene reservoir for secondary metabolism in medicinal plants.

## 1. Introduction

Seed production is one of the most important stages of plant life history. Seeds harbor high diversity of microbial taxa, known as seed-associated microbiomes, which are the endophytic or epiphytic microbial communities associated with seeds. Seed microbiomes can allow vertical transmission across generations, and have profound impacts on plant ecology, health, and productivity [1,2,3]. 

The concept of core microbiome was firstly established for human microbiome, and further expanded to other host-associated microbiomes such as plants. In addition, this concept was even used to describe microbial members shared across soils, lakes, and wastewater [4,5,6]. The composition and function of plant core microbiomes have been achieved for several model plants, such as *Arabidopsis*, maize, rice, barley, and soybean. Several studies showed that soil types and host plant genotypes are the main factors affecting the microbial community assemblage [5,7,8,9,10,11]. Studies on human microbiomes showed that human association of microbial communities have a huge impact on host metabolism [12,13,14], but few studies have analyzed the effects of plant microbiome on host metabolism. Existing plant microbiome studies have focused on rhizosphere and phyllosphere microbial communities, and our understanding of the seed microbiome has remained limited. Some studies have shown that seed core microbiome is specific for terroir and emergence [15,16]. Seed microbiomes have diverse seed–microbe interactions, and properties, such as being fast-growing, use as bio-fertilizer, antagonistic properties, and ability to cope with environmental stress [17,18,19,20,21,22,23], and are predicted to be an important biological resource for sustainable agriculture [24]. 

Danshen (*Salvia miltiorrhiza* Bge) is an important medicinal plant, mainly used to treat coronary heart diseases and cerebrovascular diseases, and has been used in China, Japan, and other east Asian countries for hundreds of years [25]. Tanshinone (a diterpenoid quinones compound) and salvianolic acid are two important active constituents of *S. miltiorrhiza*. Several studies on the associated microbes of danshen mainly focused on in vitro activities of endophytes and mycorrhizal fungi, and some endophytes could produce the similar active constituents in host plants [26,27,28,29,30,31,32,33,34]. However, the composition and function of root, leaf, or seed-associated microbiome in *S. miltiorrhiza* have not been deciphered yet.

We collected different seeds from different geographic cultivation areas and characterized the seed-associated microbiome by deep-sequencing approach to decipher the seed-associated microbiome in *S. miltiorrhiza*. Sampling was performed across the main planting zones for *S. miltiorrhiza*, including Shaanxi, Shanxi, Henan, and Shandong provinces. We used IlluminaMiSeq platform to sequence 16S ribosomal RNA (rRNA) gene, and ITS2 amplicons for DNA prepared from seven diverse geographic sources of *S. miltiorrhiza* seeds. Later, we analyzed the overlap between different source seeds and their common microbial taxa. In addition, we also analyzed the overlap in microbial taxa among danshen, maize, bean, rice, and Brassicaceae. Furthermore, we also predicted the bacterial functional profiles of core microbiome in *S. miltiorrhiza* seeds using phylogenetic investigation of communities by reconstruction of unobserved states (PICRUSt) software. 

## 2. Results

### 2.1. Genetic Diversity of Different S. miltiorrhiza Seeds

The genetic diversity of the seven *Salvia* cultivars used in this study was firstly assessed through 10 simple sequence repeat (SSR) markers (S3). According to the SSR data analyses, the individual number was suitable to represent the cultivar level of genetic diversity. At the population level, the number of different alleles (*Na*) and effective alleles (*Ne*) ranged between from 2.80 to 4.10 and 2.21 to 3.28, respectively (mean 5.305 and 3.113, respectively). This finding indicates allele differences among the *S. miltiorrhiza* groups, but these differences were insignificant. Among the population genetic diversity parameters, the ranges of Shannon’s Information Index (*I*)*,* Observed Heterozygosity (*Ho*) and Expected Heterozygosity (*He*) ranged from 0.86 to 1.24, from 0.63 to 0.85, and from 0.53 to 0.66, respectively. These results showed that the genetic diversity in different populations of *S. miltiorrhiza* was high. There were differences among the groups but the difference between groups was low. All of the observed heterozygosity (*Ho*) values were higher than expected heterozygosity (*He*), indicating the existence of significant excess heterozygosity in the population. Analysis of molecular variance (AMOVA) results indicated that the genetic diversity among cultivars was 96% much higher than genetic diversity within cultivar (4%) (Table S4). These results confirmed that the genetic variation of these *S. miltiorrhiza* populations were mainly due to the genetic differences within the population. SSR cluster analysis chart of these *S. miltiorrhiza* seeds from different geographic sources were displayed in Figure S2.

### 2.2. The Bacterial 16S rRNA and Fungal ITS Sequencing Data Set

Bacterial 16S rRNA and fungal ITS gene profiling of 21 seed samples from seven different producing area were subjected to Illumina Miseq sequencing to identify bacterial and fungal seed-associated core microbiome of cultivated *S. miltiorrhiza*. Later, bioinformatics analyses were carried out, and seed-associated core microbiome analyses were carried out among six source *S. miltiorrhiza* seeds, besides the seed of *Salvia miltiorrhiza* Bge. f. alba (a variant of *S. miltiorrhiza*) from Laiwu city in Shandong province.

The bacterial 16S rRNA sequencing resulted in 662,164 raw reads, and 662,098 of them passed the quality and length filtering. The data set comprised of 11,632–48,933 (the mean: 30,576) sequences per sample, clustered into 2548 OTUs (97%) (Table S3). The data set was rarefied, as showed in Supplementary Figure S3a. Richness estimation of seed sample complete data set revealed that Illumina 16S rDNA sequencing attained 57.0–87.4% of the estimated richness (Table S5).

The fungal ITS sequencing resulted in 1,729,121 raw reads and 1,665,477 of them passed the quality and length filtering. The data set comprised of 49,796–107,206 (the mean: 79,308) sequences per sample clustered into 222 OTUs (97%). The data set was rarefied as shown in Supplementary Figure S3b. Richness estimation of complete data set revealed that Illumina ITS sequencing attained 77.9–96.8% of estimated richness (Table S6).

### 2.3. Diversity of the Seed-Associated Bacterial Microbiome in S. miltiorrhiza

Alpha-diversity of the seed-associated bacterial microbiome of each sample was estimated using the observed species, community richness (Chao 1, expressed as the projected total number of OTU in each sample), Shannon diversity index, and evenness (Simpson’s index). The observed species, Chao 1, and Shannon diversity indices suggested that bacterial community richness showed significant difference between *S. miltiorrhiza* seed samples from different geographic origins, and community evenness showed by Simpson’s index also suggested the presence of significant differences (Tables S5 and S7, and Figure S6). DS4-LG seeds that came from Langao county, Shaanxi province had the highest community diversity within these seed samples (Shannon diversity indices: 6.63 ± 0.73).

**Figure 1:**
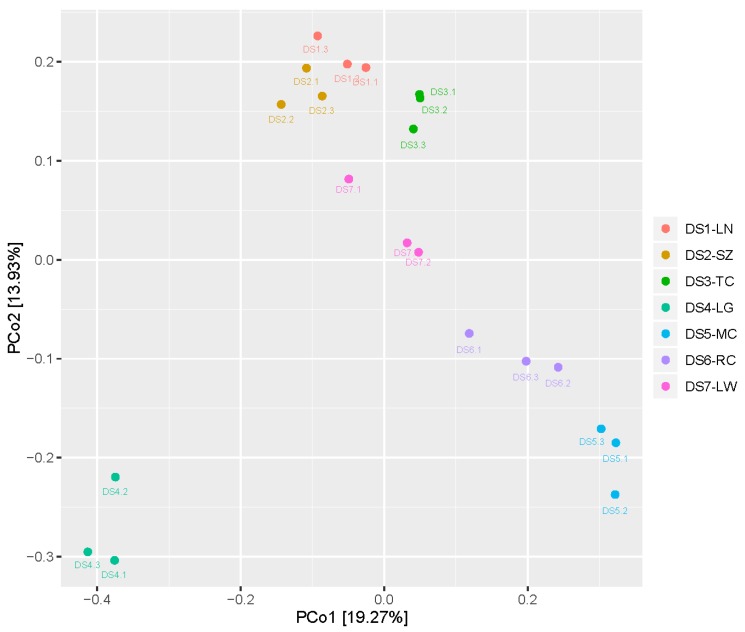
Comparison of seed-associated bacterial microbiome with cultivated area (sampling geographic origins) by principal coordinates analysis (PCoA). PCoA plot is based on unweighted unifrac distance matrix of the 16S rRNA gene amplicons. The color of the symbols indicates samples with their IDs: pale red (DS1-LG, Luonan, Shaanxi, China), light brown (DS2-SZ, Shangzhou, Shaanxi, China), light green (DS3-TC, Tongchuan, Shaanxi, China), Wathet (DS4-LG, Langao, Shaanxi, China), blue (DS5-MC, Mianchi, Henan, China), purple (DS6-RC, Ruicheng , Shanxi, China), and pink (DS7-LW, Laiwu, Shandong, China).

The variation of seed-associated microbiome diversity was explained by cultivated area (sampling location) (Figure 1). The β-diversity of the seed-associated bacterial microbiome among the different sampling locations was statistically significant. Moreover, the bacterial microbiome from different sampling locations was clustered well by unweighted unifrac distance matrix cluster analysis (Figure S1).

**Figure 2:**
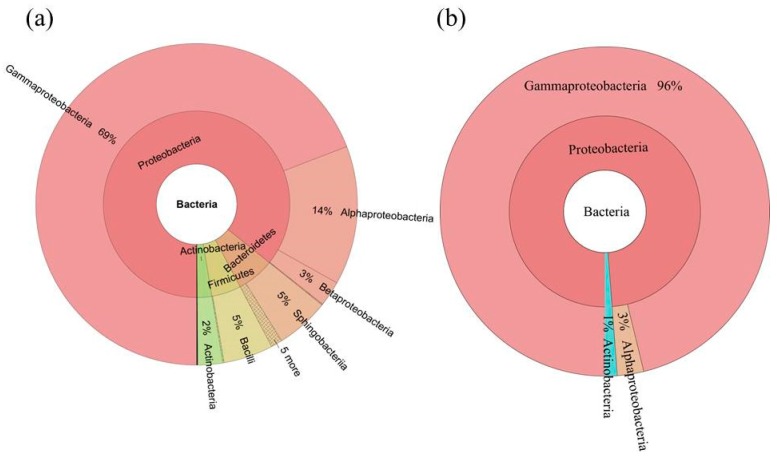
Taxonomic composition of the seed-associated (**a**) whole and (**b**) core bacterial microbiome of *S. miltiorrhiza* at the class level. Pie charts represent relative abundances of bacterial classes for the whole and core microbiome.

**Figure 3:**
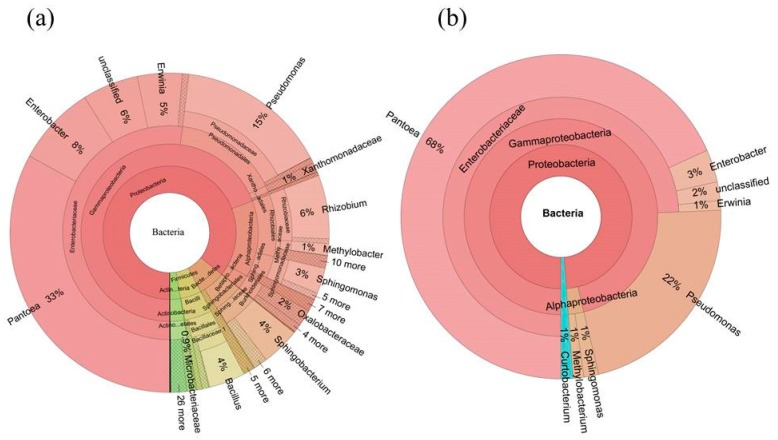
Taxonomic composition of the seed-associated (**a**) whole and (**b**) core bacterial microbiome of *S. miltiorrhiza* at the genus level. Pie charts represent relative abundances of bacterial genera for the whole and core microbiome.

Altogether, the bacterial microbiome was classified into 17 microbial phyla and candidate divisions, and 39 classes (Figure 2 and Figure 3). At phylum level, the most dominant bacterial phyla were Proteobacteria (85.9%), Bacteroidetes (6.3%), Firmicutes (4.8%), and Actinobacteria (2.9%). At class level, the bacterial microbiome was dominated by Gammaproteobacteria (67.6%), Alphaproteobacteria (15.6%), Betaproteobacteria (2.6%), Sphingobacteriia (5.0%), Bacilli (4.6%), and Actinobacteria (2.9%) (Figure 2a, Figure S4).

### 2.4. Diversity of the Seed-Associated Fungal Microbiome in S. miltiorrhiza

The fungal community richness showed significant differences between *S. miltiorrhiza* seed samples from different geographic origins, as with the bacterial microbiome, which is indicated by the observed species, Chao 1, and Shannon diversity indices. Community evenness also showed some significant differences between different seeds (Tables S6 and S8, and Figure S7).

The variation of seed-associated fungal microbiome diversity was explained by cultivated area (sampling location). The beta-diversity of the seed-associated fungal microbiome among the different sampling locations had statistical significance. However, these seed samples were not clustered well by Euclidean distance matrix, unweighted unifrac distance matrix, and weighted unifrac distance matrix.

**Figure 4:**
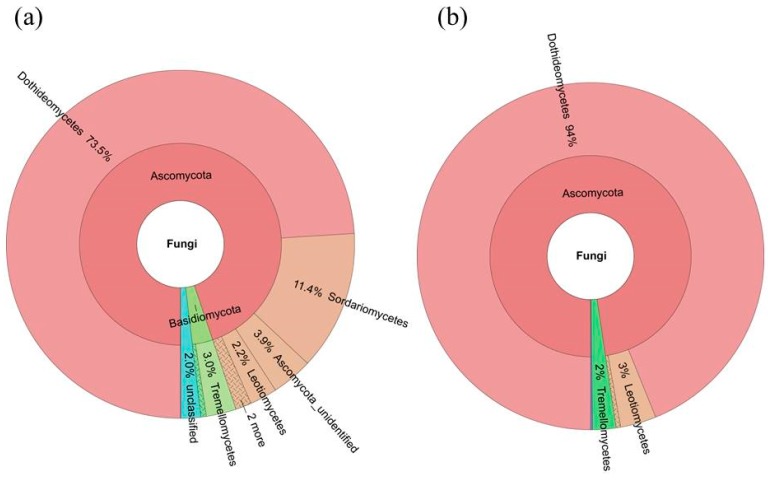
Taxonomic composition of the seed-associated (**a**) whole and (**b**) core fungal microbiome of *S. miltiorrhiza* at the class level. Pie charts represent relative abundances of fungal classes for the whole and core microbiome.

**Figure 5:**
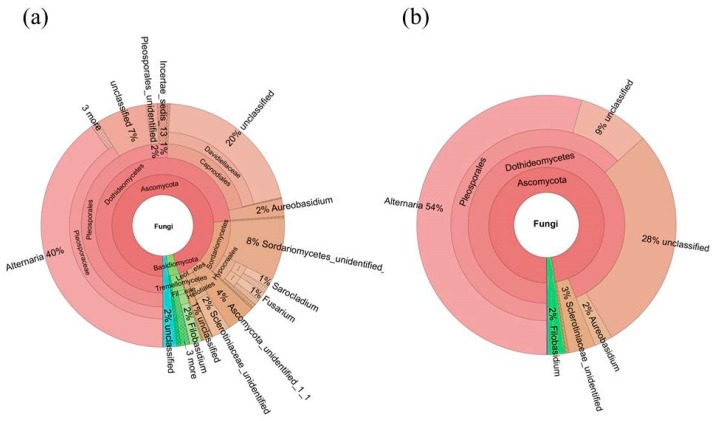
Taxonomic composition of the seed-associated (**a**) whole and (**b**) core fungal microbiome of *S. miltiorrhiza* at the genus level. Pie charts represent relative abundances of fungal genera for the whole and core microbiome.

The fungal microbiome was mainly classified into 4 phyla and candidate divisions and 19 classes, whereas 3.9% and 9.0% of the fungal microbiome remained unassigned at phylum and class level, respectively (Figure 4 and Figure 5). At the phylum level, the most dominant fungal phyla were Ascomycota (92.4%), Basidiomycota (3.6%), and other unclassified phylum (3.9%) (Figure 4, Figure S5). At the class level, fungal microbiome was dominated by Dothideomycetes (73.5%), Sordariomycetes (11.4%), Tremellomycetes (3.0%), and three other unidentified classes (two Ascomycota classes: 3.9 and 1.2%, respectively, and another unassigned class: 3.9%). 

### 2.5. Determination of the Core Bacterial Microbiome (Bacteriome) of S. miltiorrhiza Seeds

We used the persistence method to identify the OTUs present across these seed samples and determine the core bacterial microbiome (bacteriome) in *S. miltiorrhiza* seed. This core bacterial microbiome contained 16 OTUs (233,225 seq.) and corresponded to 54.5% of the whole microbiome. Taxonomic composition of the core microbiomewasmore concentrated than those of the whole microbiome at both the class and genus levels (Figure 2 and Figure 3). Gammaproteobacteria took the absolute advantage, and ran up to 96% of the core bacterial microbiome in *S. miltiorrhiza* seed at the class level. However, Alphaproteobacteria and Actinobacteria contributed 3% and 1%, respectively. At the genus level, *Pantoea* and *Pseudomonas* contained 68% and 22%, respectively. In addition, *Enterobacter* occupied 3%, whereas *Erwinia*, *Sphingomonas*, *Methylobacterium*, and *Curtobacterium* and an unclassified genus exceeded 1%. These results revealed that the seeds of *S. miltiorrhiza* shared the dominant microbiota within their microbiome (Figure 2 and Figure 3, Table S9).

### 2.6. Determination of the Core Fungal Microbiome (Mycobiome) of S. miltiorrhiza Seeds

We deciphered the core fungal microbiome (mycobiome) for *S. miltiorrhiza* seed using the same persistence approach. The core fungal microbiome of *S. miltiorrhiza* seed contained 3 OTUs (544,258 seq.) and contributed to 39.5% of the total fungal microbiome abundance in *S. miltiorrhiza* seed. At the class level, Dothideomycetes took up 94% of the core fungal microbiome in *S. miltiorrhiza* seed, and another two dominant classes were Leotiomycetes and Tremellomycetes. *Alternaria* (54%), another two unclassified genera of Dothideomycetes (28% and 9%), and a genus of Leotiomycetes (3%) were the dominant genera in the core fungal microbiome in *S. miltiorrhiza* seed, whereas *Aureobasidium* and *Filobasidium* also occupied 2% (Figure 4 and Figure 5, Table S10).

### 2.7. Predictive Function of Core Bacterial Microbiome in S. miltiorrhiza Seeds

**Figure 6:**
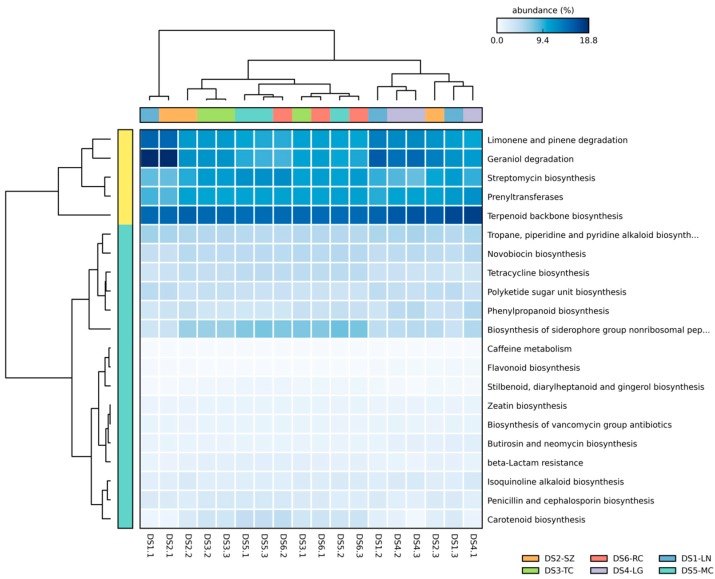
The heatmap of normalized relative abundance of imputed functional profiles of KOs assigned to biosynthesis of secondary metabolites and metabolism of terpenoids and polyketides within *S. miltiorrhiza* seed-associated core bacterial microbiome using PICRUSt grouped into level-3 functional categories.

We predicted the functional profiles of bacterial core microbiome based on the 16S rRNA gene copy number of deciphered core bacterial taxa using PICRUSt according to the KEGG Ortholog groups (KOs). We mainly focused on predicted abundances of KOs assigned to metabolism of terpenoids and polyketides, and biosynthesis of secondary metabolites. The overrepresented group included terpenoid backbone biosynthesis, limonene, pinene, and geraniol degradation, prenyltransferases in metabolism of terpenoids and polyketides, and streptomycin biosynthesis in biosynthesis of secondary metabolites. Moreover, biosynthesis of siderophore group nonribosomal peptides, tetracycline, polyketide sugar unit, tropane, piperidine, pyridine alkaloid, novobiocin, and phenylpropanoid had also a certain abundance (Figure 6). The metabolism of functional profiles was similar between different geographic origin seeds of *S. miltiorrhiza*.

## 3. Discussion

The seed-associatedmicrobiome may play an important role in plant growth and fitness by vertical transmission, influencing the primary assemblage of the plant microbiota [1,2]. Culture-independent methods using the next-generation sequencing platforms provide a high-resolution microbial community profiles for seed microbiome of maize (*Zea mays*) [35], bean (*Phaseolus vulgaris*) [15], rice (*Oryza sativa*) [36], and barley (*Hordeum vulgare*) [37], and family Brassicaceae [16] in recent years. In these studies, plant species and genotype, and growth environment were the main determinants of the seed-associated (endophyte and epiphyte) microbiota community structure. Both horizontally (acquired from the surrounding environment) and vertically (acquired directly from the parent) mode may contribute to the final composition of seed microbiome.

**Figure 7:**
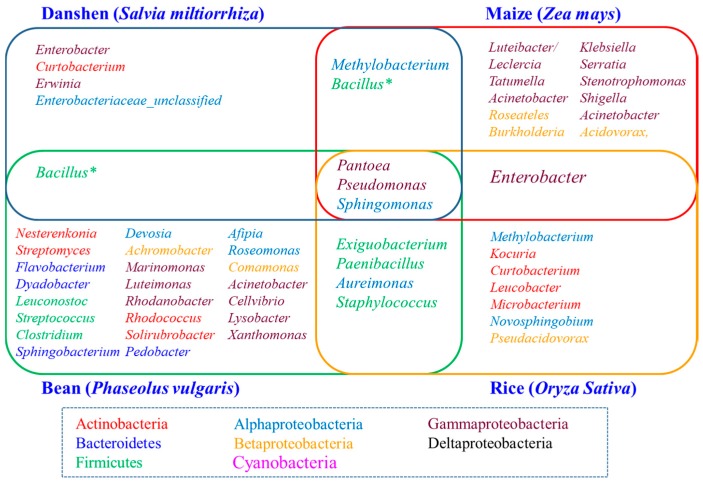
Seed-associated bacterial genera shared among danshen, maize, bean, and rice. The font color of genera was color-coded by phyla. Maize bacterial genera are based on [35]. Bean bacterial genera are based on [15]. Rice bacterial genera are based on [36]. * We isolated many *Bacillus* strains from danshen seeds, but *Bacillus* was not the dominant genus according to the 16S rRNA sequencing results. This may be due to the spore formed microbes being more easily culturable, or the bias caused by 16S rDNA primer specificity.

**Figure 8:**
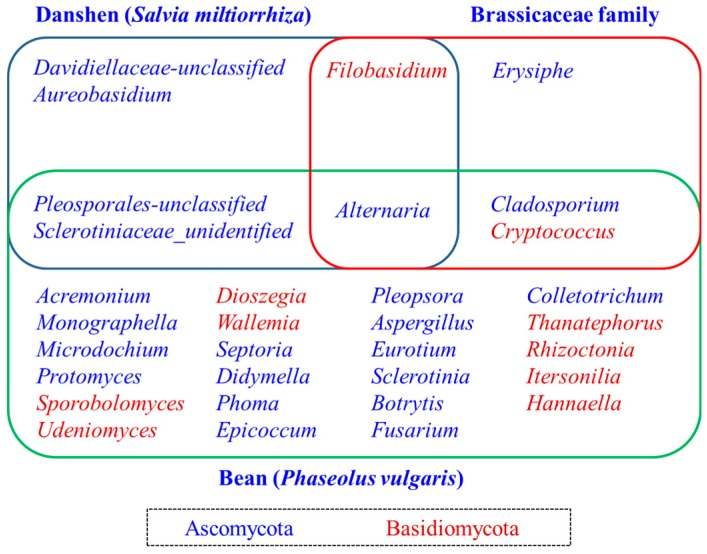
Seed-associated fungal genera shared among danshen, Brassicaceae, and bean. The font color of genera was color-coded by phyla. Brassicaceae fungal genera are based on [16]. Bean fungal genera are based on [15].

Despite significant differences between the seed microbiome of different plant species or varieties, the seed-associated microbiome consists a core set of microbial taxa. By comparing the present study of seed-associated microbiome of *S. miltiorrhiza* with maize (*Z. mays*) [35], bean (*P. vulgaris*) [15], and rice (*O. sativa*) [36], we found some enriched core taxa (genera) overlap among these plant seeds, which includethe bacterial genera *Pantoea, Pseudomonas, Sphingomonas*, and a fungal genus *Alternaria* (Figure 7 and Figure 8). A recently interesting study by Rybakova et al. confirmed that bacterial genera *Sphingomonas, Pseudomonas,* and *Bacillus* were also the most abundant taxa in the seed microbiome of *Brassica napus* [38]. These finding suggested an interesting possibility of a long association and coevolution between some seed-associated microbial taxa and their hosts. 

The predominance of *Pantoea* was especially apparent among the overlapping bacterial core taxa. In recent years, many studies have shown that some strains of *Pantoea* isolated from rice [39], maize [40], wheat [41], and *Brassica* seeds expressed a few better antagonistic activities, whereas other isolates of *Pantoea* exhibited neutral or weak pathogenic activities. Therefore, the function of *Pantoea* strains contained in seed-associated microbiome needs to be further evaluated. The other dominant genus, *Pseudomonas*, which is a kind of important plant growth-promoting bacteria (PGPR), were widely distributed in rhizosphere and endosphere of plants, and they can promote plant growth and drive root development [42,43]. *Pseudomonas* spp. represent one of the most abundant genera of the root microbiome [5,7,44,45]. Another overlapping genus, *Sphingomonas*, was also enriched taxa in some plant root systems which display plant growth promoting and bioremediation activities [46,47,48]. Therefore, the genera *Pseudomonas* and *Sphingomonas* in the seed microbiome are likely to be important reservoirs of rhizosphere or endosphere microbiome. Regarding the shared fungal core taxa, genus *Alternaria* can be a potential plant pathogen, but it also includes beneficial endophytes as biocontrol agents or other active compounds producing microbes [49,50,51,52]. Therefore, *Alternaria* can affect the germination of seeds and the assemblage of plant microbiome, and consequently, the growth and fitness of plants.

PICRUSt analysis of the seed core microbiome in *S. miltiorrhiza* showed high relative abundances of some secondary metabolism pathways or key enzymes which are closely related to terpenoid biosynthesis. Terpenoid backbone biosynthesis can provide many important precursors for terpenoid biosynthesis, which are common in upstream metabolic pathway. Limonene and pinene degradations are important component of monoterpene biosynthesis pathway. Geraniol degradation is also an important terpenoid metabolism pathway [53]. Prenyltransferases are key enzymes in many primary and secondary metabolism [54]. These pathways are common terpenoid metabolic pathways in microorganisms, and their overrepresentation in seed-associated microbiome indicates their potential for secondary metabolism gene repository in *S. miltiorrhiza.* Although the PICRUSt predicted results could not fully reflect the actual metabolic capacities of the microbial community, the seed microbiome might be considered as enriched species that are closely related to terpenoid metabolism, just as Del Giudice et al. found that the microbial community of Vetiver root involved its essential oil biogenesis [55], *S. miltiorrhiza* seed-associated microbiota might influence the secondary metabolism of host plant, and participate in the biological process of plant stress resistance and immunity.

## 4. Material and Methods

### 4.1. Sampling of Salvia miltiorrhiza Seeds

We collected 18 seeds from diverse geographic origins within the northwest of China in August 2015 to decipher core seed-associated microbiome of cultivated *S. miltiorrhiza*. We covered the main producing areas of danshen by choosing the seeds from Luonan county (34°05′26.19″ N, 110°02′6.09″ E), Shangzhou district (33°57′42.11″ N, 109°58′7.41″ E), Tongchuan city (34°54′15.02″ N, 108°57′5.61″ E), Langao county (32°18′50.68″ N, 108°54′31.81″ E) in Shaanxi province, Mianchi County (34°46′27.43″ N, 111°46′6.34″ E) in Henan province, and Ruicheng County (34°38′11.09″ N, 110°19′8.77″ E) in Shanxi province (Table S1, Figure S1). Moreover, we collected a seed of *Salvia miltiorrhiza* Bge. f. alba, a variant of *S. miltiorrhiza* from Laiwu city in Shandong province (36°12′49.00″ N, 117°40′14.86″ E), as a control of closely related species. Seeds from each geographic origin were collected as three independent replicates. All seeds were collected from *S. miltiorrhiza* standard planting base of Tasly Group Company (Tianjin, China). The quality of collected seeds was shown in Table S1. These seeds were stored separately in plastic bags at −20 °C before DNA extraction.

### 4.2. Plant DNA Exaction and SSR Genotyping

A total of 27 danshen plants were genotyped using 10 SSR molecular markers to analyze the genetic diversity existing between and within the seven *S. miltiorrhiza* cultivars (Table S2). Danshen SSR genotyping analysis method used established by Dr. Qi ZC [56,57].

Briefly, the plant seedlings were grown in a greenhouse from the seed stage and 50 mg of fresh leaf tissue were collected from each individual seedling. Genomic DNA were extracted following a modified cetyltrimethyl ammonium bromide (CTAB) protocol [58], which uses a more efficient Plant DNAzol^®^ kit (Thermo Fisher Scientific, Waltham, MA, USA). Later, DNA quality was examined on 1% agarose gel and concentration were assessed through spectrophotometry by using NanoDrop 2000 (Thermo Fisher Scientific, Waltham, MA, USA).

The SSR genotyping was carried out using 10 SSR markers covering all the species linkage groups (Table S2). PCR amplifications were performed on a T100 Thermal Cycler (Applied Biosystem, Foster City, CA, USA) with a 10 μL reaction mixture containing the following protocol: 1 μL template genomic DNA, 5 μL 2× Master Mix (TSINGKE, Hangzhou, China), 0.2 μM of each primer. The PCR protocol used was as follows: 94 °C for 3 min; followed by 35 cycles of 94 °C for 30 s, a locus-specific Ta (Table S2) for 30 s, and 72 °C 45 s, and a final extension at 72 °C for 10 min. Amplification products were checked on 2 % agarose gel stained with Gene Green Nucleic Acid dye (TIANGEN, Beijing, China).

Afterwards, PCR products were sent to TSINGKE (Hangzhou, China) where genotyped by ABI 3730 sequencer (Thermo Fisher Scientific, Waltham, MA, USA). Genetic diversity parameters, including the number of allele (*Na*), observed and expected heterozygosity (*Ho*, *He*) and polymorphism information content (PIC), which were estimated using GenAlEx 6.502 software [59]. Deviations from Hardy-Weinberg equilibrium (HWE) were tested by GENEPOP 4.2 software [60].

### 4.3. Microbial DNA Extraction

DNA from different samples was extracted using PowerPlant^®^ DNA Isolation kit (13400-50, MOBIO, Inc., Germantown, MD, USA) according to manufacturer’s instructions. Sample blanks consisted unused swabs processed through DNA extraction, and they were tested to contain no 16S amplicons. The total DNA was eluted in 50 µL of elution buffer by a modification of the procedure described by the manufacturer (MOBIO), and stored at −80 °C until measurement in the PCR by LC-Bio Technology Co., Ltd., Hangzhou, China.

### 4.4. PCR Amplification, 16S rDNA or ITS Sequencing and Data Analysis

We amplified the V3–V4 region of the bacterial 16S rRNA gene and ITS2 region of the eukaryotic (fungal) small-subunit rRNA gene using the total DNA from 21 *S. miltiorrhiza* seed samples as a template and the primer (319F 5′-ACTCCTACGGGAGGCAGCAG-3′; 806R 5′-GGACTACHVGGGTWTCTAAT-3′) for bacterial microbiota and primer (ITS7F 5′-ACTCCTACGGGAGGCAG CAG-3′; ITS4R 5′-GGACTACHVG GGTWTCTAAT-3′) for fungal microbiota. The 5′ ends of the primers were tagged with specific barcodes per sample and sequencing universal primers.

All reactions were carried out in 25 µL (total volume) mixtures containing approximately 25 ng of genomic DNA extract, 12.5 µL PCR Premix, 2.5 µL of each primer, and PCR-grade water to adjust the volume. PCR reactions were performed in a master cycler gradient thermocycler (Eppendorf, Hamburg, Germany) set to the following conditions: initial denaturation at 98 °C for 30 s; 35 cycles of denaturation at 98 °C for 10 s, annealing at 54/52 °C for 30 s, and extension at 72 °C for 45 s; and then a final extension at 72 °C for 10 min. The PCR products were confirmed with 2% agarose gel electrophoresis. Throughout the DNA extraction process, ultrapure water instead of a sample solution, was used to exclude the possibility of false-positive PCR results as a negative control. The PCR products were normalized by AxyPrep TM Mag PCR Normalizer (Axygen Biosciences, Union City, CA, USA), which allowed the skipping of the quantification step, regardless of the PCR volume submitted for sequencing. The amplicon pools were prepared for sequencing with AMPure XT beads (Beckman Coulter Genomics, Danvers, MA, USA), and the size and quantity of the amplicon library were assessed on the LabChip GX (Perkin Elmer, Waltham, MA, USA) and with the Library Quantification Kit for Illumina (Kapa Biosciences, Woburn, MA, USA), respectively. PhiX Control library (V3) (Illumina) was combined with the amplicon library (expected at 30%). The library was clustered to a density of approximately 570 K/mm^2^. The libraries were sequenced either on 300PE MiSeq runs, and one library was sequenced with both protocols using the standard Illumina sequencing primers, which eliminated the need for a third (or fourth) index read.

Samples were sequenced on an Illumina MiSeq platform according to the manufacturer’s recommendations provided by LC-Bio. Paired-end reads were assigned to samples based on their unique barcode, and truncated by cutting off the barcode and primer sequence. Paired-end reads were merged using PEAR (v.0.9.6) (Heidelberg, Germany) [61]. Quality filtering on the raw tags was performed under specific filtering conditions to obtain the high-quality clean tags according to the FastQC (v.0.10.1) (New Delhi, India) [62]. Chimeric sequences were filtered using VSEARCH (v.2.3.4) (Oslo, Norway) and sequences with ≥97% similarity were assigned to the same operational taxonomic units (OTUs) using the same software [63]. Representative sequences were chosen for each OTU, and taxonomic data were then assigned to each representative sequence using the RDP (Ribosomal Database Project) classifier. To examine the differences of the dominant species in different groups, multiple sequence alignments were conducted using PyNAST (v.1.2) software (Boulder, CO, USA) [64] to study phylogenetic relationships of different OTUs. Abundance information of OTUs was normalized using a standard of sequence number corresponding to the sample with the least sequences. Alpha diversity was applied in analyzing complexity of species diversity for a sample through 4 indices, including Chao 1, Shannon, Simpson, and Observed species. All these indices in our samples were calculated with QIIME software (Boulder, CO, USA) in Python (v.1.8.0) (La Jalla, CA, USA) [65]. Beta diversity analysis was used to evaluate differences of samples in species complexity. Beta diversity was calculated by principle coordinates analysis (PCoA) and cluster analysis by QIIME [66].

### 4.5. Determination of Core Microbiome of S. miltiorrhiza Seed

Metagenomics Core Microbiome Exploration Tool (MetaCoMET) was applied to decipher the core microbiome across cultivated *S. miltiorrhiza* seeds according to the membership and persistence methods, especially by focusing on the latter methods [67]. In brief, we input the OUT BIOM file generated by QIIME, which described the above and metadata file in turn. We uploaded these files to the website, and selected parameters and Venn type. Later, we submitted them to the web platform to obtain results. 

### 4.6. Predict Microbial Functional Profiles of Core Microbiome

PICRUSt software (http://picrust.github.io/picrust) was used to predict the microbial functional profiles of core microbiome in *S. miltiorrhiza* seeds. We modified the sequence data format according to the platform requirements, and then performed the functional prediction using the method provided by PICRU St. Briefly, the OUT BIOM table of seed-associated core microbiome was used as an input file for metagenome imputation of *S. miltiorrhiza* seed samples, and predicted gene class abundances were analyzed at KEGG Orthology group levels 3 [68]. Results from PICRUSt were analyzed in statistical analysis of taxonomic and functional profiles (STAMP) [69].

## 5. Conclusions

In conclusion, deciphering the core microbiome across different cultivated *S. miltiorrhiza* seeds indicated that seed microbiome is a distinctive genetic resource for the host plant. Although some studies had indicated seed microbiomes have significant impacts on host plant health and productivity [1,2,16,17,18], our study provides the first insights into the seed-associated core microbiome of a medicinal plant. Our PICRUSt prediction analysis revealed that these microbial core taxa can influence the growth and quality of *S. miltiorrhiza*. Especially, we found the seed-associated microbiome could be a reservoir and supplement of secondary metabolic capabilities, in addition to the host plant genome. Just as Aleti et al. found, the secondary metabolite genes encoded by potato rhizosphere microbiomes were diverse and vary with the different samples and vegetation stage, which influence on the growth and metabolism of the host plant [70]. Our study suggested that some core taxa of seed microbiome not only promoted seed germination and plant growth, but also regulated and participated in the secondary metabolism of host plants.

## Supplementary Materials

Supplementary materials can be found at http://www.mdpi.com/1422-0067/19/3/672/s1.
