# Supplementary material for: Core Microbiome of Medicinal Plant Salvia miltiorrhiza Seed: A Rich Reservoir of Beneficial Microbes for Secondary Metabolism?
Source: Int J Mol Sci. 2018 Feb 27;19(3):672. doi: 10.3390/ijms19030672 (PMC5877533; doi:10.3390/ijms19030672)
Supplement: Supplementary file 1 [file ijms-19-00672-s001.pdf]

Supplementary Material

Figure S1 Geographic distribution of *Salvia miltiorrhiza* seeds Sampling sites.

Figure S2 SSR cluster analysis chart of *S.miltiorrhiza* seeds from different geographic sampling sites.

Figure S3 Rarefaction curves showing the observed OTU richness (97% identity).

Figure S4 Taxonomic composition of seed-associated bacterial microbiome of *S.miltiorrhiza* at class level.

Figure S5 Taxonomic composition of seed-associated fungal microbiome of *S.miltiorrhiza* at class level.

Figure S7 Fungal community diversity statistics for *S. miltiorrhiza* seed samples.

Table S1 Sampling sources and quality of *S. miltiorrhiza* seeds.

Table S2 Characterization of 10 microsatellite loci List.

Table S3 SSR genetic diversity indexes.

Table S4 AMOVA results.

Table S5 Summary of sequencing and statistical data of bacterial microbiome of *S. miltiorrhiza* seeds.

Table S6 Summary of sequencing and statistical data of fungal microbiome of *S. miltiorrhiza* seeds.

Table S7 Alpha-diversity and richness estimates indices for bacterial 16S rDNA amplicon libraries of *S. miltiorrhiza* seed samples.

Table S8 Alpha-diversity and Richness estimates indices for Fungal ITS amplicon libraries of *S. miltiorrhiza* seed samples.

Table S9 Top 8 most abundant bacterial genera within core bacterial microbiome of *S. miltiorrhiza* seed.

Table S10 Top 8 most abundant fungal genera within core fungal microbiome of *S. miltiorrhiza* seed.

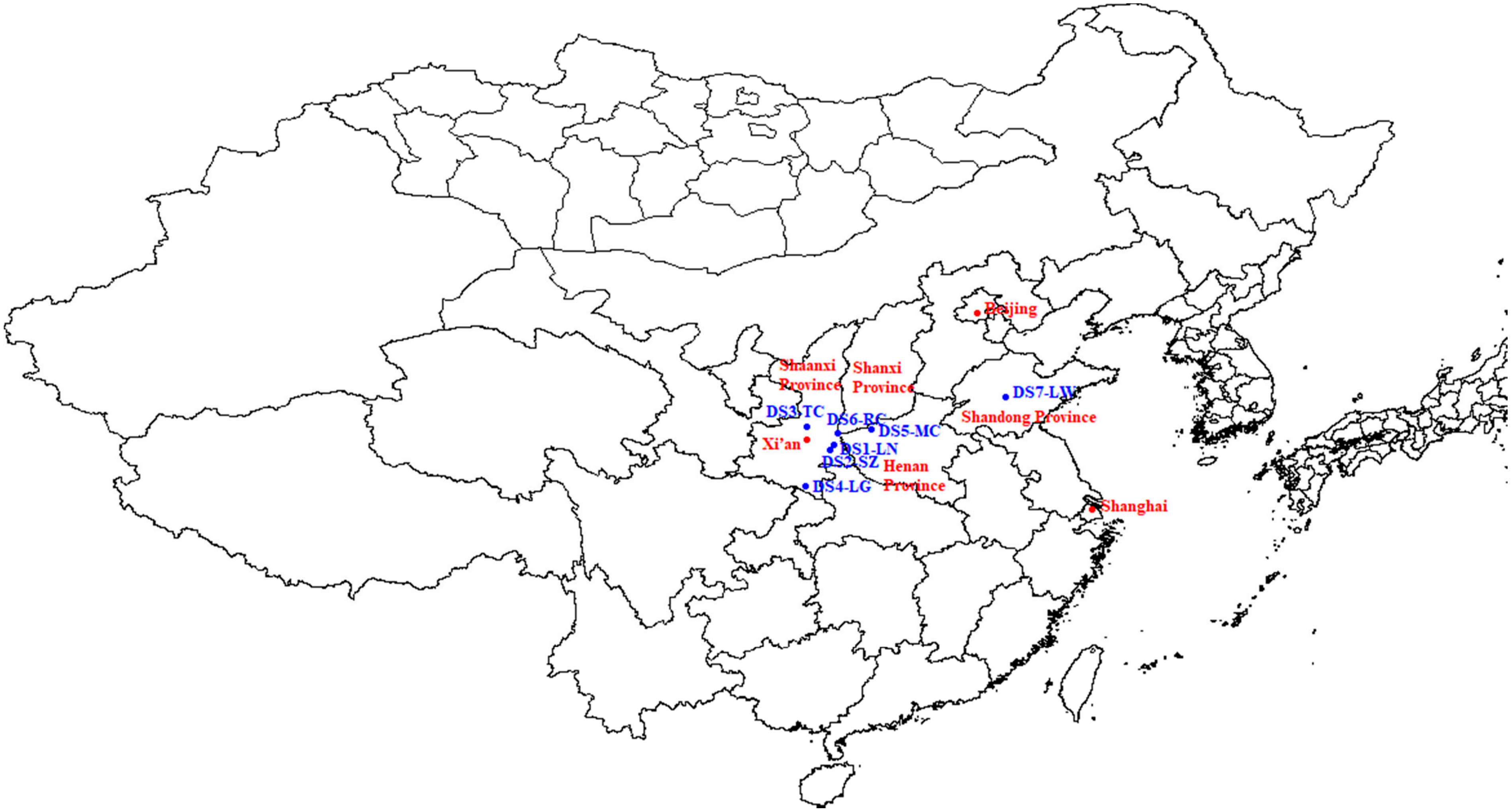

Figure S1 Geographic distribution of *Salvia miltiorrhiza* seeds Sampling sites.

The symbols indicates sample with their IDs and their sampling sites, DS1-LG (Luonan County, Shaanxi Province), DS2-SZ (Shangzhou District, Shaanxi Province), DS3-TC (Tongchuan City, Shaanxi Province), DS4-LG (Langao County, Shaanxi Province), DS5-MC (Mianchi County, Henan Province), DS6-RC (Ruicheng County, Shanxi Province), DS7-LW (Laiwu County, Shandong Province). This map was generated from the free website DIVA-GIS (http://www.diva-gis.org/) and modified by their free soft DIVA-GIS 7.5 (http://www.diva-gis.org/download).

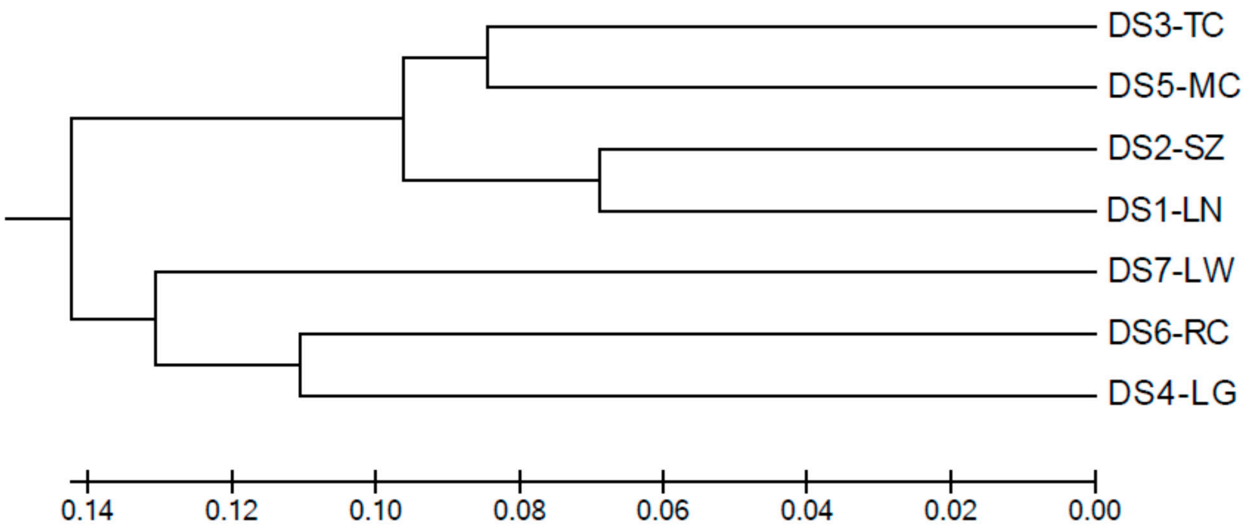

Figure S2 SSR cluster analysis chart of *S.miltiorrhiza* seeds from different geographic sampling sites.

DS1-LG, Luonan County, Shaanxi Province, DS2-SZ, Shangzhou District, Shaanxi Province, DS3-TC, Tongchuan City, Shaanxi Province, DS4-LG, Langao County, Shaanxi Province, DS5-MC, Mianchi County, Henan Province, DS6-RC, Ruicheng County, Shanxi Province, DS7-LW, Laiwu County, Shandong Province.

(a)

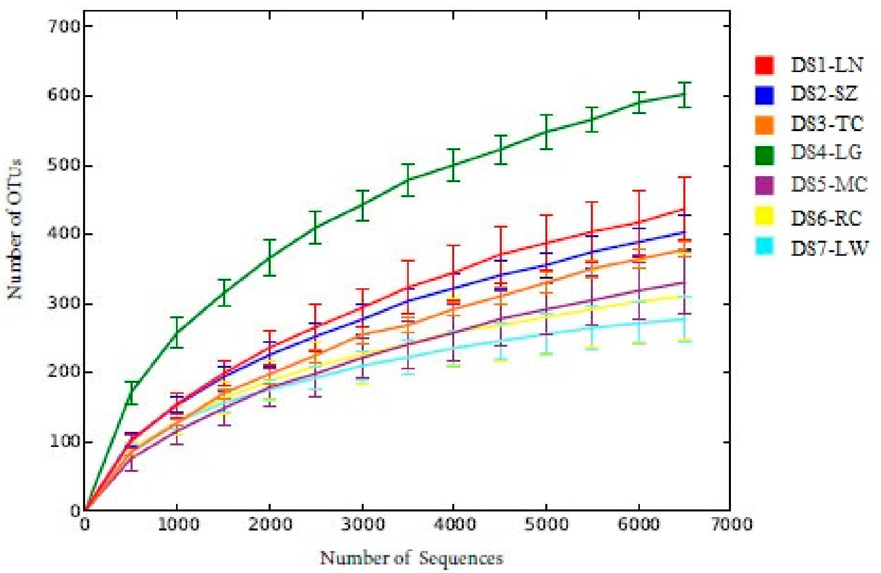

(b)

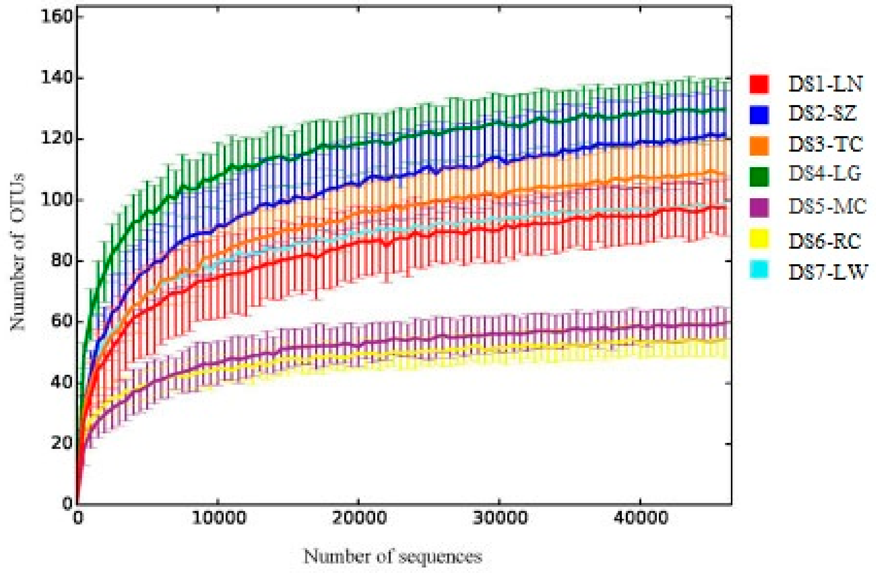

Figure S3 Rarefaction curves showing the observed OTU richness (97% identity).

The curves are grouped according to the sample sites. (a) Rarefaction curves for bacterial 16S rRNA gene amplicon libraries. (b) Rarefaction curves for fungal ITS2 region of small-subunit rRNA gene amplicons.

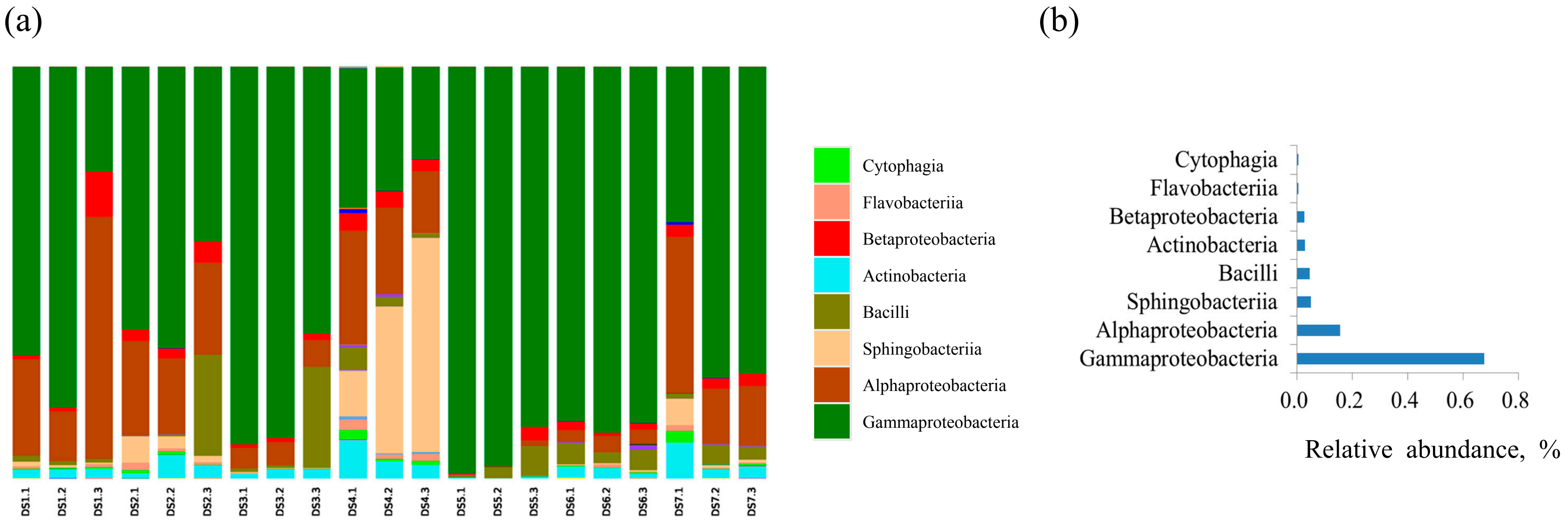

Figure S4 Taxonomic composition of seed-associated bacterial microbiome of *S.miltiorrhiza* at class level.

(a) Bar charts represent relative abundance of every *S.miltiorrhiza* seed samples at class level. (b)For the whole microbiome, main classes relative abundance shown as separate diagram.

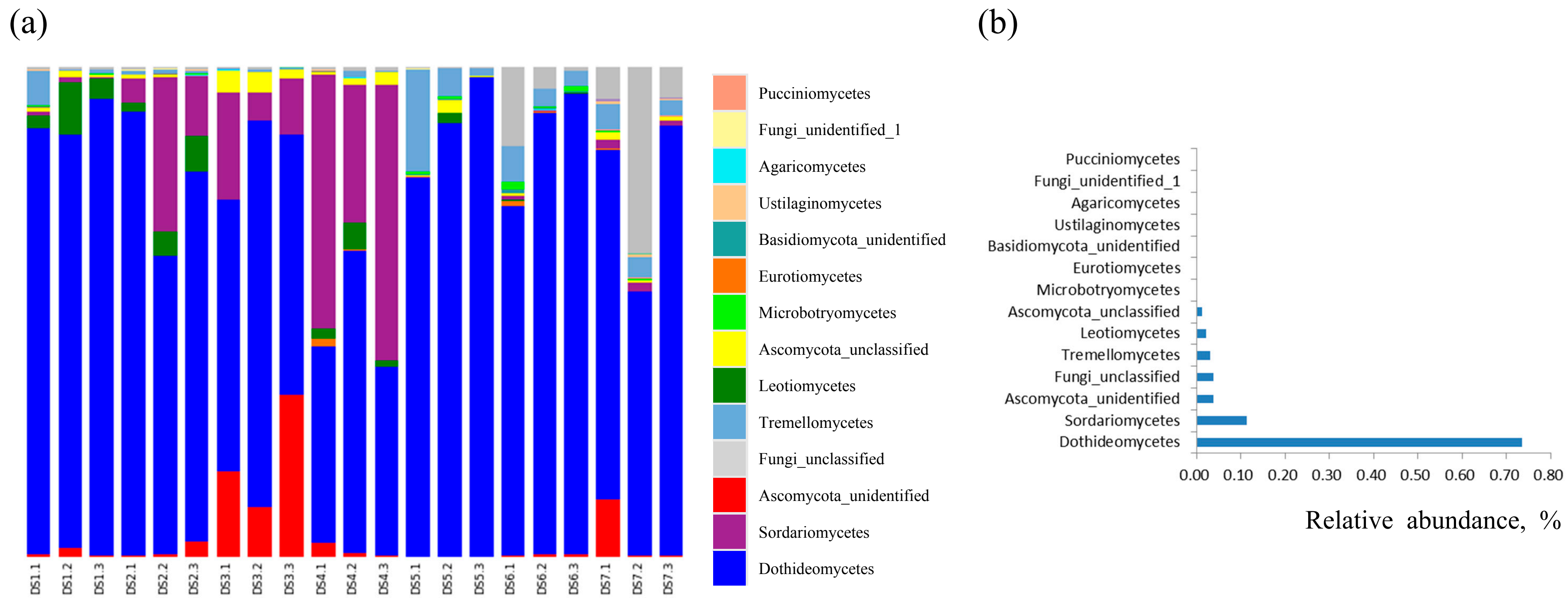

Figure S5 Taxonomic composition of seed-associated fungal microbiome of *S.miltiorrhiza* at class level.

(a) Bar charts represent relative abundance of every *S.miltiorrhiza* seed samples at class level. (b)For the whole microbiome, main classes relative abundance shown as separate diagram.

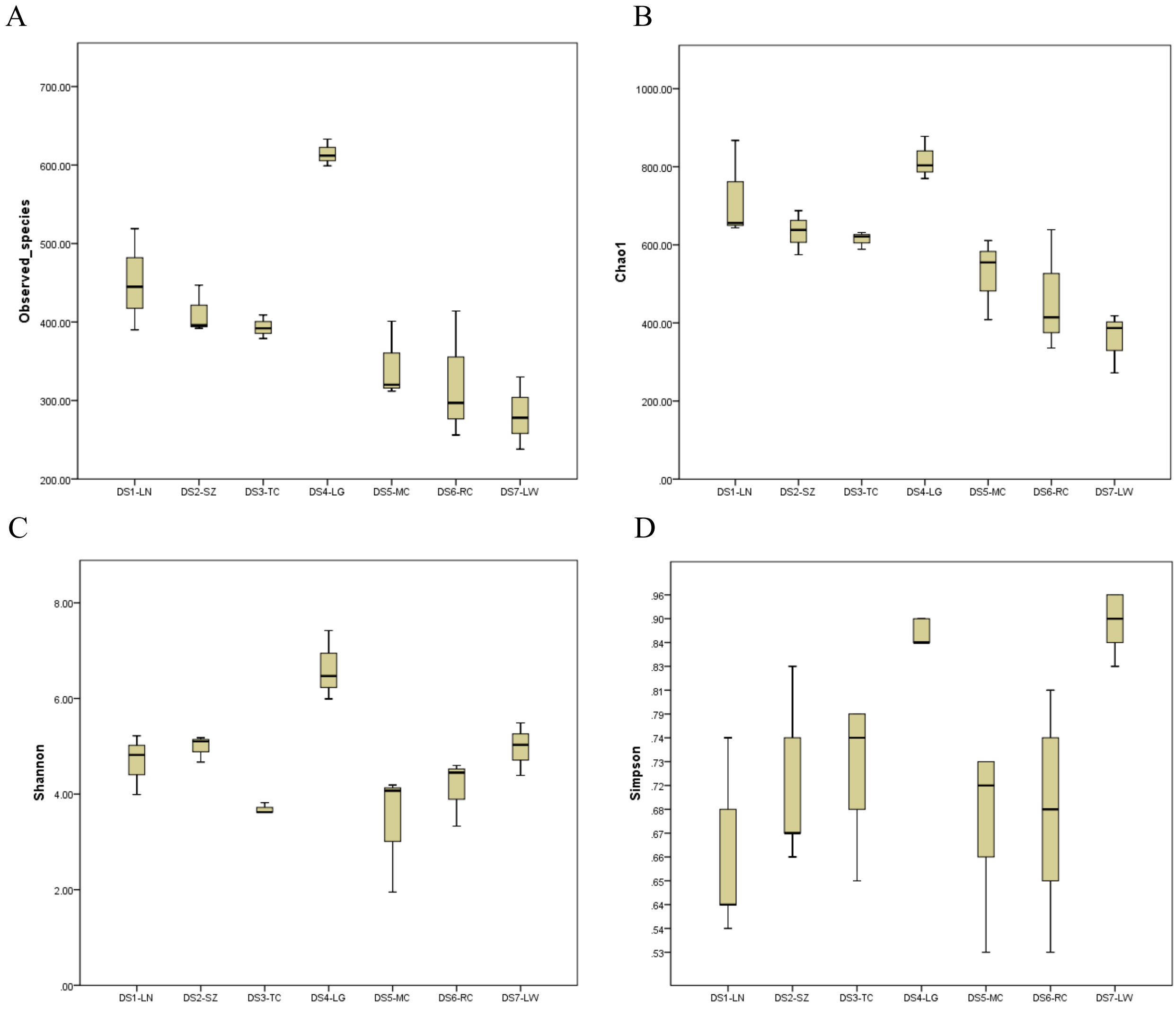

Figure. S6 Bacterial community diversity statistics for *S. miltiorrhiza* seed samples.

(A) Observed species. (B) Chao1 index, which indicated the bacterial community richness (expressed as the projected total number of OTU in each sample). (C) Shannon index (H'), which is a combined measure of bacterial community richness and evenness. (D) Simpson's index, which measures bacterial community evenness. Error bars represent the 95% confidence intervals of the data. The observed species, Chao1 and Shannon diversity indicated that bacterial community richness showed significant difference between *S.miltiorrhiza* seeds samples from different geographic origins, and bacterial community evenness showed by Simpson's index also revealed some significant differences (Table S3 Supporting information).

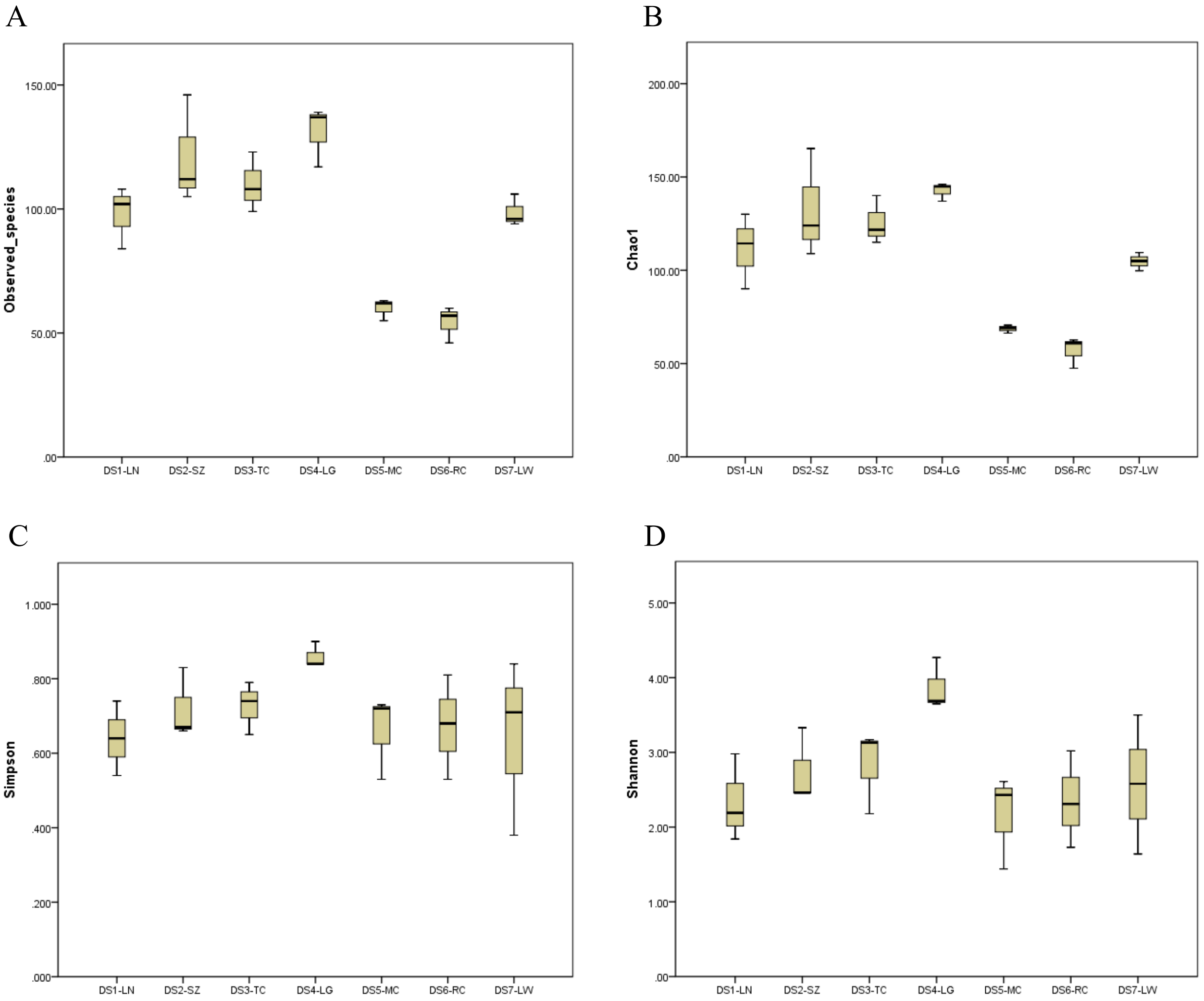

Figure S7 Fungal community diversity statistics for *S. miltiorrhiza* seed samples.

(A) Observed species. (B) Chao1 index, which indicated the fungal community richness (expressed as the projected total number of OTU in each sample). (C) Shannon index (H'), which is a combined measure of fungal community richness and evenness. (D) Simpson's index, which measures fungal community evenness. Error bars represent the 95% confidence intervals of the data. The observed species, Chao1 and Shannon diversity indicated that fungal community richness showed significant difference between *S.miltiorrhiza* seeds samples from different geographic origins, and fungal community evenness showed by Simpson's index also revealed some significant differences (Table S3 Supporting information).

Table S1 Sampling sources and quality of *S. miltiorrhiza* seeds.

Sample ID	Source	Latitude / longitude	Germination percentage (%)	Thousand kernel weight (g)	Moisture Content (%)	Viability (%)
DS1-LN	Luonan county, Shaanxi province	34°05′ 26.19″ N, 110°02′ 6.09″ E	86.33±1.20a	2.03±0.02a	9.00±0.07b	84.00±3.46a
DS2-SZ	Shangzhou district,Shaanxi province	33°57′ 42.11″ N, 109°58′ 7.41″ E	70.00±3.05b	1.97±0.02ab	7.88±0.01c	82.00±6.92a
DS3-TC	Tongchuan city, Shaanxi province	34°54′ 15.02″ N, 108°57′ 5.61″ E	48.00±6.11c	1.87±0.03bc	9.64±0.02a	57.33±11.56bc
DS4-LG	Langao county, Shaanxi province	32°18′ 50.68″ N, 108°54′ 31.81″ E	41.33±7.53cd	1.79±0.02c	9.51±0.05ab	50.67±13.28bc
DS5-MC	Mianchi county, Henan province	34°46′ 27.43″ N, 111°46′ 6.34″ E	43.67±0.33cd	1.67±0.03d	8.89±0.02b	40.67±10.97c
DS6-RC	Ruicheng county, Shanxi province	34°38′ 11.09″ N, 110°19′ 8.77″ E	31.67±4.17d	1.81±0.07bc	9.33±0.04ab	52.67±2.40bc
DS7-LW*	Laiwu county, Shandong province	36°12′ 49.00″ N, 117°40′ 14.86″ E	ND	ND	ND	ND

Abbreviations: ND – not determined.

*DS7-LW is a seed of *Salvia miltiorrhiza* Bge. f. alba, a variant of *S. miltiorrhiza.*

Table S2 Characterization of 10 microsatellite loci List.

Locus	Primer sequence(5'-3')	motif	Product size(bp)	T_a (°C)	N_a	H_O	H_E	PIC	Genbank accession no.
P017	F:CAGAGGGAGAAAGGAGAAAT<br>R:GTATGAGCCCAAGTTCAATC	(AG)6	164	53	7	0.703	0.701	0.638	KY400593
P018	F:CTAATGTGTCCAAACCACCT<br>R:TACGGGATGTTTGTTTCTCC	(ATT)6	157	53	9	0.547	0.817	0.784	KY400594
P025	F:ACGCAAACGTCTCCTTATC<br>R:GTGGAGCTCAGGAATAAGTG	(TCG)7	148	54	6	0.344	0.581	0.524	KY400597
P028	F:GGTTGCAGTGCCATATAGTT<br>R:GTCGTCTCCCATCAACTTC	(GTT)9	145	54	10	0.75	0.854	0.829	KY400599
P036	F:GAGAGTATCGCAAACGAGTC<br>R:CATGCATTCTTCACACAGAC	(AT)9	133	54	8	0.531	0.726	0.684	KY400604
P040	F:AAAGATCAGCCACGTATCAC<br>R:ATTATGCTGCACCGATCTAC	(AAATCA)4	132	53	6	0.703	0.635	0.595	KY400605
P052	F:GTCAGTGTTCAGACATCCTATG<br>R:AACTGAGCCATCAACATCTC	(TGA)9	110	55	8	0.594	0.695	0.654	KY400612
P074	F:TACGACAAGCAAGACTACAGC<br>R:ATTACCCAAGCCTCCAAG	(CAG)7	187	54	6	0.5	0.536	0.499	KY400620

P079	F:GCTGCAGTGGAAGAATATGT<br>R:TCCATTCCAACTCATCTCTG	(GA)9	127	53	11	0.547	0.832	0.809	KY400623
P083	F:GTGATAACGACCAAAGAGGT<br>R:GCTCTCTCTCGCATCCTTAT	(GA)7	100	54	8	0.766	0.675	0.625	KY400625

T_a Annealing temperature of primer pairs, N_a number of alleles, H_o observed heterozygosity, H_E expected heterozygosity; PIC polymorphism information content

Significant deviations from Hardy–Weinberg equilibrium at *$P < 0.05$, **$P < 0.01$, and ***$P < 0.001$, respectively.

Table S3 SSR genetic diversity indexes.

Cultivar	*Na*†	*Ne*†	*I*†	*Ho*†	*He*†	*P*†
TC	2.80±0.249	2.21±0.153	0.86±0.071	0.83±0.084	0.53±0.031	0.34±0.311
SZ	2.80±0.200	2.37±0.167	0.91±0.070	0.85±0.076	0.56±0.034	0.36±0.274
SM	3.10±0.379	2.49±0.221	0.95±0.126	0.63±0.125	0.55±0.066	0.47±0.328
RC	4.10±0.379	3.28±0.304	1.24±0.107	0.80±0.050	0.66±0.041	0.48±0.191
LW	2.90±0.233	2.32±0.121	0.92±0.064	0.78±0.079	0.56±0.022	0.48±0.243
LN	2.90±0.277	2.39±0.252	0.89±0.119	0.65±0.100	0.53±0.066	0.45±0.302
AK	3.70±0.335	2.63±0.287	1.06±0.114	0.73±0.079	0.57±0.053	0.62±0.293
Total Mean	3.19±0.123	2.53±0.090	0.98±0.039	0.77±0.033	0.57±0.018	0.46±0.281

Mean diversity indexes of the seves studied *Salvia miltiorrhiza* varieties calculated on the SSR dataset.

† mean ± standard error

Table S4 AMOVA results.

Source	df	SS	MS	Est. Var.	%	*P*
Among cultivars	6	23.250	3.875	0.168	4%	<0.001
Within cultivars	28	105.000	3.750	3.750	96%	<0.001

Analysis of molecular variance (AMOVA) showing the partitioning of genetic variation within and between varieties (df = degree of freedom, SS = sum of squares, MS mean squares, Est. var. = estimate of variance, % = percentage of total variation, P is based on 9999 permutations)

Table S5 Summary of sequencing and statistical data of bacterial microbiome of *S. miltiorrhiza* seeds.

Sample	Raw Data		Valid Data		Valid%	Q20%	Q30%	GC%	observed_species	shannon	simpson	chao1
	Tag	Base	Tag	Base								
DS1.1	30627	14.46M	29721	12.22M	97.04	94.89	85.07	53.18	390	3.99	0.75	655.94
DS1.2	29203	13.78M	28356	11.72M	97.10	94.72	84.47	54.64	519	5.22	0.91	867.43
DS1.3	30986	14.63M	30107	12.15M	97.16	95.17	85.69	53.24	445	4.82	0.86	643.45
DS2.1	36203	17.16M	35684	14.58M	98.57	96.68	89.74	52.84	396	5.18	0.91	574.89
DS2.2	43953	20.83M	43145	17.81M	98.16	96.41	89.16	54.20	447	5.1	0.87	638.11
DS2.3	31440	14.84M	30557	12.61M	97.19	94.84	84.88	53.59	392	4.67	0.89	687.55
DS3.1	25543	12.06M	24755	10.35M	96.92	94.28	83.53	55.05	409	3.62	0.65	631.58
DS3.2	27233	12.85M	26463	11.06M	97.17	94.44	83.93	54.87	392	3.62	0.69	588.91
DS3.3	28703	13.55M	27822	11.62M	96.93	94.46	84.00	54.28	379	3.82	0.78	621.35
DS4.1	30164	14.24M	29192	12.02M	96.78	94.66	84.47	53.25	633	7.42	0.98	803.50
DS4.2	12151	5.76M	11632	4.81M	95.73	93.10	79.13	53.13	612	6.47	0.94	877.80
DS4.3	49745	23.58M	48933	20.24M	98.37	96.28	88.72	53.23	599	5.99	0.89	769.85
DS5.1	29720	14.03M	27066	11.43M	91.07	91.46	77.46	55.98	320	1.95	0.36	555.17
DS5.2	28595	13.50M	27490	11.62M	96.14	94.15	83.09	55.74	312	4.19	0.84	408.67

DS5.3	35350	16.69M	33980	14.34M	96.12	94.27	83.62	55.47	401	4.07	0.74	611.04
DS6.1	27698	13.13M	27116	11.40M	97.90	95.73	87.39	54.70	297	4.45	0.81	414.44
DS6.2	25133	11.91M	24233	10.17M	96.42	95.91	87.84	55.26	256	3.33	0.64	335.60
DS6.3	35612	16.81M	34448	14.36M	96.73	94.49	84.11	55.33	414	4.60	0.83	638.64
DS7.1	43052	20.41M	42344	17.49M	98.36	96.43	89.15	53.6	238	5.49	0.96	272.22
DS7.2	27948	13.19M	27044	11.32M	96.77	94.47	84.13	53.89	278	4.39	0.83	386.90
DS7.3	33105	15.63M	32010	13.37M	96.69	94.67	84.44	54.92	330	5.03	0.90	418.39

Table S6 Summary of sequencing and statistical data of fungal microbiome of *S. miltiorrhiza* seeds.

Sample	Raw Data		Valid Data		Valid%	Q20%	Q30%	GC%	observed_species	shannon	simpson	chao1
	Tag	Base	Tag	Base								
DS1.1	109371	51.62M	107206	25.97M	98.02	99.74	98.93	51.67	108.00	2.98	0.74	130.00
DS1.2	82606	38.99M	81800	19.81M	99.02	99.78	99.04	51.31	84.00	2.19	0.64	90.07
DS1.3	86351	40.76M	84486	20.18M	97.84	99.79	99.12	51.74	102.00	1.84	0.54	114.36
DS2.1	74134	35.14M	73428	17.54M	99.05	99.82	99.29	53.59	105.00	2.46	0.66	108.93
DS2.2	78038	36.99M	77144	18.73M	98.85	99.72	99.02	56.91	146.00	3.33	0.83	165.25
DS2.3	90553	42.74M	90231	21.48M	99.64	99.73	98.96	56.70	112.00	2.46	0.67	124.00
DS3.1	60322	28.47M	58653	14.77M	97.23	99.46	98.13	57.41	123.00	3.13	0.79	140.10
DS3.2	93459	44.30M	93243	23.12M	99.77	99.69	98.92	53.40	108.00	3.17	0.74	115.00
DS3.3	75824	35.79M	75343	19.46M	99.37	99.42	98.04	57.91	99.00	2.18	0.65	121.67
DS4.1	98566	46.52M	96057	23.62M	97.45	99.59	98.54	60.21	117.00	3.69	0.84	137.00
DS4.2	89719	42.53M	87938	21.04M	98.01	99.75	99.12	55.97	137.00	4.27	0.90	144.86
DS4.3	81017	38.40M	80607	19.55M	99.49	99.73	99.02	56.83	139.00	3.65	0.84	146.00
DS5.1	49827	23.52M	49796	12.79M	99.94	98.23	94.06	50.57	62.00	2.43	0.73	69.00
DS5.2	70388	33.22M	67527	16.62M	95.94	99.67	98.70	51.64	63.00	2.61	0.72	66.33
DS5.3	76073	35.91M	75474	18.41M	99.21	99.73	98.88	52.24	55.00	1.44	0.53	70.60
DS6.1	129523	61.39M	106958	28.12M	82.58	99.29	97.87	53.91	60.00	3.02	0.81	60.86
DS6.2	70922	33.48M	63684	15.59M	89.79	99.66	98.76	52.56	57.00	2.31	0.68	62.60
DS6.3	71557	33.77M	58418	14.30M	81.64	99.68	98.82	52.08	46.00	1.73	0.53	47.50
DS7.1	94304	44.70M	92766	22.52M	98.37	99.70	98.95	54.98	96.00	3.50	0.84	99.75
DS7.2	77144	36.41M	75668	18.37M	98.09	99.68	98.79	54.14	94.00	2.58	0.71	105.00
DS7.3	69423	32.77M	69050	17.18M	99.46	99.62	98.56	51.53	106.00	1.64	0.38	109.46

Table S7 Alpha-diversity and richness estimates indices for bacterial 16S rDNA amplicon libraries of *S. miltiorrhiza* seed samples.

Sample ID	observed_species	shannon	simpson	chao1	Coverage, %
DS1-LN	451.33±64.73b	4.67±0.63bc	0.64±0.10b	722.27±125.86ab	62.83±5.52b
DS2-SZ	411.67±30.66bc	4.98±0.27b	0.72±0.10ab	633.52±56.47bc	65.33±7.24b
DS3-TC	393.33±15.04bc	3.69±0.12c	0.73±0.07ab	613.95±22.28bc	64.13±2.86b
DS4-LG	614.67±17.16a	6.63±0.73a	0.86±0.03a	817.05±55.24a	75.43±4.99ab
DS5-MC	344.33±49.24c	3.40±1.26c	0.66±0.11b	524.96±104.51bc	66.50±9.38b
DS6-RC	322.33±81.99c	4.13±0.69bc	0.67±0.14b	462.89±157.22c	70.93±5.79ab
DS7-LW	282.00±46.13c	4.97±0.55b	0.90±0.07a	359.17±76.93c	79.40±7.76a

Table S8 Alpha-diversity and Richness estimates indices for Fungal ITS amplicon libraries of *S. miltiorrhiza* seed samples.

Sample ID	observed_species	shannon	simpson	chao1	Coverage, %
DS1-LN	98.00±12.19b	2.34±0.58b	0.64±0.10b	111.48±20.12b	88.53±5.13a
DS2-SZ	121.00±21.93a	2.75±0.50b	0.72±0.10ab	132.73±19.16ab	91.70±4.18a
DS3-TC	110.00±12.12ab	2.82±0.56b	0.72±0.07ab	125.59±13.00ab	87.70±6.25a
DS4-LG	131.00±12.16a	3.87±0.35a	0.86±0.03a	142.62±4.90a	91.73±5.49a
DS5-MC	60.00±4.36c	2.16±0.63c	0.66±0.11b	68.64±2.16c	87.60±8.78a
DS6-RC	54.33±7.37c	2.35±0.65c	0.67±0.14b	56.99±8.26c	95.50±3.92a
DS7-LW	98.66±6.43b	2.57±0.93b	0.64±0.24b	104.74±4.86b	94.17±4.05a

Table S9 Top 8 most abundant bacterial genera within core bacterial microbiome of *S. miltiorrhiza* seed*.

Genus ID	Taxonomy	Relative abundance, %
1	d__Bacteria; p__Proteobacteria; c__Gammaproteobacteria; o__Enterobacteriales; f__Enterobacteriaceae; g__*Pantoea*	68%
2	d__Bacteria; p__Proteobacteria; c__Gammaproteobacteria; o__Pseudomonadales; f__Pseudomonadaceae; g__*Pseudomonas*	22%
3	d__Bacteria; p__Proteobacteria; c__Gammaproteobacteria; o__Enterobacteriales; f__Enterobacteriaceae; g__*Enterobacter*	3%
4	d__Bacteria; p__Proteobacteria; c__Gammaproteobacteria; o__Enterobacteriales; f__Enterobacteriaceae; g__unclassified	2%
5	d__Bacteria; p__Proteobacteria; c__Alphaproteobacteria; o__Sphingomonadales; f__Sphingomonadaceae; g__*Sphingomonas*	1%
6	d__Bacteria; p__Proteobacteria; c__Alphaproteobacteria; o__Rhizobiales; f__Methylobacteriaceae; g__*Methylobacterium*	1%
7	d__Bacteria; p__Actinobacteria; c__Actinobacteria; o__Actinomycetales; f__Microbacteriaceae; g__*Curtobacterium*	1%
8	d__Bacteria; p__Proteobacteria; c__Gammaproteobacteria; o__Enterobacteriales; f__Enterobacteriaceae; g__*Erwinia*	1%

*abundant bacterial genus means relative abundance great than 1%.

Table S10 Top 8 most abundant fungal genera within core fungal microbiome of *S. miltiorrhiza* seed*.

<table>
<tr><th>Genus ID</th><th>Taxonomy</th><th>Relative abundance, %</th></tr>
<tr><td>1</td><td>d__Fungi; p__Ascomycota; c__Dothideomycetes; o__Pleosporales; f__Pleosporaceae; g__Alternaria</td><td>54%</td></tr>
<tr><td>2</td><td>d__Fungi; p__Ascomycota; c__Dothideomycetes; o__Capnodiales; f__Davidiellaceae; g__unclassified</td><td>28%</td></tr>
<tr><td>3</td><td>d__Fungi; p__Ascomycota; c__Dothideomycetes; o__Pleosporales; f__unclassified; g__unclassified</td><td>9%</td></tr>
<tr><td>4</td><td>d__Fungi; p__Ascomycota; c__Leotiomycetes; o__Helotiales; f__Sclerotiniaceae; g__Sclerotiniaceae_unidentified</td><td>3%</td></tr>
<tr><td>5</td><td>d__Fungi; p__Ascomycota; c__Dothideomycetes; o__Dothideales; f__Dothioraceae; g__Aureobasidium</td><td>2%</td></tr>
<tr><td>6</td><td>d__Fungi; p__Basidiomycota; c__Tremellomycetes; o__Filobasidiales; f__Filobasidiaceae; g__Filobasidium</td><td>2%</td></tr>
<tr><td>7</td><td>d__Fungi; p__Ascomycota; c__unclassified; o__unclassified; f__unclassified; g__unclassified</td><td rowspan="2">about 2%</td></tr>
<tr><td>8</td><td>d__Fungi; p__unclassified; c__unclassified; o__unclassified; f__unclassified; g__unclassified</td></tr>
</table>

*abundant bacterial genus means relative abundance great than 1%.
